# Effect of Plastein Reaction on Physical and Chemical Characteristics of Corn Glutelin Peptides and Quality of Chiffon Cake

**DOI:** 10.3390/foods14193392

**Published:** 2025-09-30

**Authors:** Yang Sun, Wan-Ying Zhang, Chun-Li Song, Zhi-Qin Pan, Guo-Jun Du, Zhi-Qiang Song, Jian Ren, Li-Ying Bo, Jing-Jing An, Meng Wang

**Affiliations:** 1 Faculty of Food Quality and Safety, Qiqihar University, Qiqihar 161006, China; xinyang_1021@163.com (Y.S.); 18745150721@163.com (W.-Y.Z.); songchunlilily@sina.com (C.-L.S.);; 2 Tianjin Institute of Industrial Biotechnology, Chinese Academy of Sciences, Tianjin 300308, China

**Keywords:** corn glutelin hydrolysate, plastein reaction, functional properties, baked products, textural properties

## Abstract

Corn glutelin hydrolysate (CGH) was prepared by alkaline protease hydrolysis of corn glutelin and further modified by histidine (His) and tryptophan (Trp) through the Plastein reaction, obtaining His-fortified CGH (His-CGH) and Trp-fortified CGH (Trp-CGH). The functional properties (solubility, foaming capacity, and emulsifying activity) of the modified peptides were analyzed. The corresponding modifiers were added to baked products to evaluate potential application in the baking field. The effects of the modifiers on batter density, specific volume, and textural properties of chiffon cake were investigated. This study aimed to enhance the functional characteristics of corn glutelin and provide a theoretical basis for the development of functional products or green food additives. Corn glutelin hydrolysate supplemented with His-CGH and Trp-CGH exhibited improved solubility, foaming stability, and emulsifying capacity. Compared with CGH, the foamability (FC) of Trp-CGH increased by 9%, the foaming stability (FS10) at 10 min elevated by 8.41%, the foaming stability (FS20) at 20 min improved by 14.79%, and the foaming stability at 30 min (FS30) raised by 14.14%. The emulsifying activity of Trp-CGH improved by 10.65 m^2^/g, and the emulsifying stability increased by 10.57 min. Furthermore, the batter density of the cake sample with Trp-CGH decreased by 0.028 g/cm^3^, the specific volume increased by 0.29 cm^3^/g, the baking loss rate lowered by 0.99%, and the hardness reduced by 0.36 N. The improvement of these quality indexes remarkably enhanced the sensory acceptance and texture of the cake sample. Overall, it also reveals that the addition of the Plastein reaction modifiers before baking also highlights their potential as green food additives in baking products.

## 1. Introduction

With the improvement in living standards, consumer demands for food quality are also increasing day by day. Many chemically synthesized food additives, such as synthetic pigments and nitrites, are associated with varying degrees of health risks, including potential carcinogenic effects [1,2]. Therefore, the development of healthy and eco-friendly food additives has become a major focus in food science. Cereals are rich sources of dietary fiber, vitamins, minerals, and biologically active ingredients, playing an essential role in promoting health [3]. For instance, black rice contains flavonoids and polyphenols, which exhibit strong antioxidant and anti-inflammatory properties [4]. Wheat oligopeptides are rich in glutamic acid and hydrophilic amino acid residues, which enable them to interact with starch and water molecules in flour-based products. As a result, they can serve as natural food additives to enhance the rheological properties and gluten network structure, thereby improving the texture and sensory qualities of flour products [5]. Natural green food additives are mainly derived from natural ingredients such as plant extracts and microbial fermentation products. Such additives not only avoid the possible health risks of synthetic additives, but also bring unique quality improvement to food [6]. For example, natural pigments such as carotene and anthocyanins can give food a bright color, while natural antioxidants such as vitamin C and tea polyphenols can effectively delay the oxidative deterioration of food [7]. Furthermore, a previous study has demonstrated that rice protein hydrolysate could inhibit the interaction between gluten proteins and starch found in wheat flour, thus heightening the sensory quality of crisp biscuits [8]. Therefore, cereals and their processed derivatives containing a variety of bioactive components have attracted increasing attention in the food industry.

Maize is a cereal crop widely cultivated in many regions worldwide and serves as a primary raw material for the production of starch, starch sugars, ethanol, and other industrial products [9]. Corn gluten meal (CGM), a by-product obtained from corn kernels after starch or ethanol extraction, is rich in various bioactive nutrients, including 62–71% protein, 20% starch, 10% cellulose, and vitamin A, making it a valuable natural source of food nutrients [9,10]. The proteins found in CGM can be classified into albumin, globulin, gliadin, and glutenin. Among them, corn glutelin exhibits poor solubility, being insoluble in water or alcohol and only soluble in dilute acid or alkali solutions, which significantly limits its application in the food industry. In order to broaden its application scope, it is necessary to enhance the solubility and other functional properties of corn glutelin. Protein hydrolysis is an effective method for improving functional properties, because protease can decompose corn gluten into small peptides or amino acids, resulting in improved solubility [11,12]. However, bioactive peptides are relatively unstable and susceptible to environmental factors that may compromise their functional activity and thermal stability. Therefore, it is key to explore safe and effective strategies to enhance their functional performance of active peptides. The Plastein reaction can alter the amino acid sequence and three-dimensional structure of proteins or peptides, and the reagents used in this reaction are food-grade, making it suitable for modifying bioactive peptides and strengthening the functional and nutritional properties of proteins [13,14]. Studies have indicated that the Plastein reaction can significantly improve the physicochemical properties of proteins. For instance, it has been reported that the Plastein reaction can increase the antioxidant capacity of casein hydrolysates. Compared with casein hydrolysates, casein hydrolysates modified by phenylalanine or tyrosine via the Plastein reaction exhibited obviously increased DPPH radical scavenging activity (increased by 10.5% and 8%, respectively) [15]. Another study also expounded that the bitter taste of salmon skeleton protein hydrolysates was reduced through the Plastein reaction, while both physicochemical and antioxidant properties were simultaneously enhanced [16]. Current research indicates that the Plastein reaction involves enzyme-catalyzed cleavage of proteins into smaller peptides or amino acids with enhanced surface activity, thereby improving foaming capacity and foam stability [17]. It is worth mentioning that this reaction can markedly increase the surface hydrophobicity of proteins, enhance their adsorption capacity at the gas–liquid interface, and consequently improve foam formation and stability.

Histidine is an essential amino acid that plays multiple critical roles in human health [18,19]. It participates in protein synthesis, is a major component of the active site of some enzymes, with unique physiological functions and many benefits for human health [20]. Tryptophan is another essential amino acid that serves as a precursor for the synthesis of proteins and a range of important bioactive molecules. A deficiency in tryptophan can lead to a variety of health issues, primarily affecting neurotransmitter synthesis, metabolic processes, and immune system function [21,22].

Currently, research on corn-derived peptides primarily focuses on antihypertensive peptides, high-F-ratio oligopeptides, and sobering peptides. In contrast, studies on corn glutelin hydrolysates modified via the Plastein reaction remain limited. In this study, corn glutelin was hydrolyzed using alkaline protease, and the resulting peptides were further modified through the Plastein reaction with exogenous amino acids (histidine and tryptophan). The antioxidant activity and functional properties of the modified peptides were evaluated; meanwhile, the effects of corn glutelin peptides modified by the Plastein reaction on cake quality were assessed. The main purpose of this work is to improve the physicochemical properties of corn glutelin hydrolysate and develop its new application in the baking field.

## 2. Materials and Methods

### 2.1. Materials and Reagents

Corn gluten meal (CGM) was obtained from Heilongjiang Beidahuang Group (Harbin, China). Alkaline protease (activity: 20,000 U/g) was purchased from Solarbio Science and Technology Co., Ltd. (Beijing, China). L-histidine (His) and L-tryptophan (Trp) were obtained from Macklin Biochemical Technology Co., Ltd. (Shanghai, China). Whole milk powder was sourced from Beidahuang Wandashan Dairy Co., Ltd. (Harbin, China). Flour used to prepare the cake sample was acquired from Xinxiang Liangrun Whole Grain Food Co., Ltd. (Xinxiang, China). All other chemicals, such as sodium chloride and ethanol, were of analytical grade and obtained from commercial sources.

### 2.2. Extraction of Corn Glutelin and Preparation of Its Hydrolysates

The extraction of corn glutelin was performed according to the method of Zheng et al. [23], with minor modifications. Corn gluten meal was mixed with 0.02 mol/L phosphoric acid solution (pH 6.5) at a ratio of 1:10 (*w*/*v*), followed by the addition of 1% (*w*/*v*) α-amylase (3700 U/g) to hydrolyze at 70 °C for 2 h. The pH was maintained between 5.5 and 7.5 throughout the entire reaction period using 0.1 mol/L NaOH. The enzyme was inactivated by heating in a boiling water bath for 15 min, then cooled to room temperature and centrifuged at 4000 rpm for 10 min. The resulting precipitate was washed three times with deionized water, dried, and ground into powder. This powder was thoroughly mixed with absolute ethanol at a solid–liquid ratio of 1:10 (*w*/*v*), stirred at room temperature for 2 h, and centrifuged at 4000 rpm for 15 min. The precipitate was re-extracted following the same procedure, and these samples were naturally dried and ground into powder. Subsequently, the powder was mixed with 70% ethanol at a ratio of 1:10 (*w*/*v*), stirred at 60 °C for 2 h, and centrifuged at 4000 rpm for 10 min. The final precipitate was naturally dried and ground to obtain the crude glutelin powder.

The prepared corn gluten meal was extracted with 0.1 mol/L NaOH solution (1:10, *w*/*v*) at 60 °C for 2 h, then centrifuged at 4000 rpm for 15 min. The supernatant was adjusted to pH 4.8 with 4 mol/L HCl and then centrifuged again. The resulting precipitate was washed three times with 70% ethanol, followed by two washes with distilled water adjusted to pH 4.8. A small amount of water was added to dissolve the precipitate, and the pH was subsequently adjusted to 8.0 with 2 mol/L NaOH solution. After freeze-drying for 48 h, corn glutelin (CG) was obtained.

The prepared corn glutelin was added to distilled water (pH 8.0) to form a protein suspension with a substrate concentration of 5 g/100 mL. Subsequently, 3% alkaline protease (2.0 × 10^5^ U/g) was added to hydrolyze the protein at 50 °C for 8 h, with the pH maintained at 8.0 during the reaction period using 1 mol/L NaOH. After centrifugation at 4500 rpm for 20 min, the supernatant was collected and lyophilized to obtain the corn glutelin hydrolysate (CGH).

### 2.3. Preparation of Modifiers of Plastein Reaction

The Plastein reaction was carried out at 45 °C for 7 h, while adding substrate concentration of 40% corn glutelin protein hydrolysate (*w*/*w*) and 1% alkaline protease (2.0 kU/g). The addition ratio of exogenous amino acids was set to 0.7 mol/mol free amino acids. At the end of the reaction, the enzyme was inactivated by heating in a water bath at 95 °C for 15 min. The resulting modified product was freeze-dried and stored for subsequent use.

### 2.4. Determination of Free Amino Content and Hydrolysis Degree

The free amino acid content and hydrolysis degree of the sample were determined using the OPA method [24]. The OPA reagent was prepared as follows: 80 mg of OPA was weighed and dissolved in 2 mL of absolute ethanol in the dark. Subsequently, 1.9068 g of sodium tetraborate, 0.1 g of SDS, and 0.088 g of DTT were dissolved in water. The solutions were combined and diluted to a final volume of 100 mL in a brown volumetric flask:

$$Y=\frac{A\times N}{131.17\times X}$$

Y: Free amino content (mmol/g); A: Leucine concentration (µg/mL); N: Dilution factor; X: Protein mass per unit volume (g/L); 131.17: Molecular weight of leucine (g/mol).

$$\mathrm{DH}(\%)=\frac{Y_{2}-Y_{1}}{h_{\mathrm{tot}}}\times 100\%$$

Y_1_: Content of free amino groups when not hydrolyzed (mmol/L); Y_2_: Free amino acid content of protein after hydrolysis (mmol/L); h_tot_: Gram equivalents of peptide bonds per gram of protein (Corn Protein 8.38).

### 2.5. Determination of Antioxidant Activity of Modifiers

The DPPH radical scavenging activity was determined using the method described by Musa et al. [25]. A sample solution with a concentration of 1 mg/mL was prepared. In total, 100 μL of the sample was added to a 96-well plate, followed by the addition of 100 μL of DPPH free radical solution in absolute ethanol. The mixture was allowed to react for 30 min in the dark, and the absorbance at 517 nm was measured as A_i_. In another 96-well plate, 100 μL of the sample was mixed with 100 μL of absolute ethanol, and the absorbance was recorded as A_j_. For the control, 100 μL of absolute ethanol was reacted with 100 μL of DPPH free radical solution in absolute ethanol, and the absorbance was measured as A_0._ All measurements were performed in sextuplicate.

$$\mathrm{DPPH}\;\mathrm{Scavenging}\;\mathrm{rate}\;(\%)=[1-\frac{A_{i}-A_{j}}{A_{0}}]\times 100\%$$

The ABTS radical scavenging activity was measured according to the method of Stella et al. [26], with slight modifications. In total, 40 μg/mL of ABTS free radical stock solution was prepared and diluted with PBS to an absorbance of 0.7 ± 0.02 at 734 nm, serving as the ABTS free radical working solution. A sample solution with a concentration of 1 mg/mL was prepared, and 100 μL of the sample solution was added to a 96-well plate, followed by the addition of 100 μL of ABTS free radical working solution. The resulting absorbance was recorded as A_i_. Phosphate-buffered saline (PBS) was used instead of the sample to serve as the control, with the absorbance recorded as A_0_. The mixture was vortexed and left to stand in the dark for 10 min; afterwards, the absorbance value was measured at 734 nm. All measurements were repeated six times.

$$\mathrm{ABTS}\;\mathrm{Scavenging}\;\mathrm{rate}\;(\%)=[\frac{A_{0}-A_{i}}{A_{0}}]\times 100\%$$

### 2.6. Determination of Physicochemical Properties of Modifiers

#### 2.6.1. Solubility Determination

The Folin phenol method was employed to determine the soluble protein content according to the method provided by Zengin et al. [27], with slight modifications. Solution A was prepared by dissolving 2 g of Na_2_CO_3_ in 100 mL of 0.1 mol/L NaOH solution, and solution B was prepared by dissolving 0.5 g of CuSO_4_·5H_2_O crystals in 100 mL of 1% potassium sodium tartrate solution. The Folin phenol reagent A solution was prepared at a ratio of 50:1 immediately before use. Deionized water with pH values of 2.0, 3.0, 4.0, 5.0, 6.0, 7.0, 8.0, 9.0, and 10.0 was prepared using 0.1 mol/L sodium hydroxide and 0.1 mol/L hydrochloric acid solutions. Sample solutions with a concentration of 0.5 mg/mL were prepared using deionized water at different pH levels. After centrifugation, the supernatant was collected, and the absorbance was measured.

$$Solubility\left(\%\right)=\frac{\mathrm{Supernatant}\;\mathrm{protein}\;\mathrm{content}}{Total\;protein\;content}\times 100$$

#### 2.6.2. Determination of Emulsification and Emulsification Stability

Based on the method of Ghribi [28], with slight modifications, a sample solution with a protein concentration of 2 mg/mL was prepared. It was mixed with soybean oil at a volume ratio of 3:1 and homogenized for 2 min using a high-shear machine at 14,000 rpm. An aliquot of 50 μL of emulsion was withdrawn from the bottom of the centrifuge tube at 0 and 30 min, respectively. The sample was diluted 100-fold with 0.1% SDS solution, thoroughly mixed, and the absorbance at 500 nm was measured using the SDS solution as a blank. The formulas for determining emulsifying activity (EAI) and emulsifying stability (ESI) are as follows:

$$\mathrm{EAI}=\frac{2\times 2.303\times A_{0}\times \mathrm{DF}}{C\times \varphi \times \theta \times 10,000}$$

$$\mathrm{ESI}=\frac{A_{0}\times 30}{A_{0}-A_{30}}$$

DF: Dilution factor, 100; C: Protein concentration, g/mL: *φ*: Optical path, 1 cm; *θ*: Oil volume fraction, 0.25; A_0_: Absorbance of emulsion volume at 0 min; A_30_: Absorbance of emulsion volume at 30 min.

#### 2.6.3. Determination of Foaming and Foaming Stability

The method of Chen et al. [29] was referenced and modified accordingly. A 1% solution was prepared in a 50 mL centrifuge tube and homogenized at 10,000 rpm for 2 min. The resulting foam volume was recorded as the foaming capacity. After standing for 10, 20, and 30 min, respectively, the foam volume was measured each time and recorded as foam stability. All experiments were conducted in triplicate.

$$\mathrm{FA}(\%)=\frac{H_{1}}{H_{0}}\times 100\%$$

$$\mathrm{FS}(\%)=\frac{H_{2}}{H_{1}}\times 100\%$$

H_0_: Initial height (cm); H_1_: 0 initial foam height after homogenization (cm); H_2_: Foam height at 10 min (cm); H_3_: Foam height at 20 min (cm); H_4_: Foam height at 30 min (cm).

### 2.7. Determination of the Quality of Chiffon Cake After Adding Modifiers

#### 2.7.1. The Preparation of Chiffon Cake

First, weigh 30.00 g of egg yolk, add 20.00 g of white granulated sugar, and stir until throughly mixed. In total, 50.00 g of milk and 50.00 g of corn oil were stirred and emulsified to form a milky white mixture, which was then added to the whipped yolks. In total, 90.00 g of low-gluten flour was sifted into the mixture and gently incorporated. Meanwhile, 50.00 g of white granulated sugar and 1% of modifier were added to the 90.00 g egg white, which were then whipped to stiff peaks and carefully folded into the batter. The final batter was gently poured into a mold and baked at 150 °C for approximately 35 min. After baking, the cake was inverted and cooled at room temperature for 1 h, then placed into a sealed plastic bag to prevent moisture loss.

#### 2.7.2. Determination of Density of Cake Batter

Batter density was determined according to the relative density method described by Baik et al. [30]. The formula for calculating batter density is as follows:

$$\rho =1\times \frac{M_{2}-M_{0}}{M_{1}-M_{0}}$$

M_0_, M_1_, and M_2_ are the mass of the flat-bottomed container, the mass of the flat-bottomed container filled with clear water, and the mass (g) of the flat-bottomed container filled with batter, respectively; ρ is the density of cake batter, g/cm^3^; 1 is the density of clear water, g/cm^3^.

#### 2.7.3. Determination of Baking Characteristics of Cakes

After baking, the cake was cooled at room temperature for 1 h, and its mass was measured using an electronic balance. The cake volume was determined by the rapeseed displacement method, and the specific volume was subsequently calculated [31]. The baking loss rate was calculated based on the mass difference between the sample before and after baking [32].

$$\mathrm{SV}=\frac{V}{M}$$

$$\mathrm{BL}(\%)=\frac{M_{1}-M}{M_{1}}\times 100\%$$

SV: cake specific volume (cm^3^/g); BL: baking loss rate (%); V: cake volume (cm^3^); M: cake mass (g); M_1_: batter mass (g).

#### 2.7.4. Analysis of Internal Structure of Cake Crumb

After baking, the cake was cooled at room temperature for 2 h, and the crumb structure was analyzed. A 10 cm^2^ slice was taken from the center of the cake sample following different treatments. A high-resolution image was captured using a high-pixel camera, converted to 8-bit format, and threshold processing was automatically performed using the Otsu algorithm in ImageJ software (ImageJ.Ink) to obtain the following parameters: pore area, pore density, and porosity [33].

#### 2.7.5. Determination of Texture Characteristics of Cakes

The baked cake was stored at room temperature for 2 h and then sliced into uniform pieces with a thickness of 1.5 cm. Texture analysis was conducted using a texture analyzer equipped with a P/36 R probe under the following conditions. The speed is 1 mm/s, the trigger force is 0.05 N, the interval between two compressions is 5 s, and the testing height is 50 mm. A full texture profile analysis (TPA) was performed to determine the hardness, chewiness, springiness, and adhesiveness of the cake.

#### 2.7.6. Determination of Cake Color

The three key color parameters of both the cake crust and the inner core of the cut slices were measured using a portable Chroma Meter. These parameters included L (lightness), a (redness/greenness), and b (yellowness/blueness). Each sample was measured at three different points, and the average value was calculated.

#### 2.7.7. Sensory Measurement Evaluation of Cake Samples

The sensory evaluation of the chiffon cake sample was carried out by providing three coded samples, one of which was a control sample, and the other two were cake samples with His-CGH and Trp-CGH, which were tasted by trained panellists. Reviewers evaluated five attributes: appearance, color, flavor, taste, and overall acceptability. The order of presentation of the samples was randomly coded using a three-digit number. Pure drinking water was provided to clean the mouth during the assessment. Sensory evaluation was performed according to the scoring criteria provided by Podder et al. [34].

### 2.8. Statistical Analysis of Data

All experimental data were independently repeated three times (n = 3), and the results were expressed as mean ± standard deviation (SD). One-way analysis of variance (ANOVA) was conducted using SPSS 27.0 software, and statistical significance was determined at *p* < 0.05. Data processing and image analysis were performed with Excel 2011, GraphPad Prism 8.0.2, and ImageJ, respectively.

## 3. Results and Discussion

### 3.1. DH and Free Amino Group Content of All Samples

The free amino group contents of all samples are summarized in Table 1. The free amino group content of CGH prepared after hydrolysis was 1.7928 ± 0.0170 mmol/g protein, which obviously increased by 0.0231 ± 0.0013 mmol/g protein (*p* < 0.05), compared to CG itself, and the corresponding hydrolysis degree (DH) reached 21.12%. During the process of protein hydrolysis, peptide bonds are cleaved, and the amino groups that were previously involved in peptide bond formation are released, leading to an increase in the level of free amino groups [35].

Compared with the physical mixtures (His+CGH and Trp+CGH), the free amino group content of these modifiers (His-CGH and Trp-CGH) was significantly decreased (*p* < 0.05) by 0.3799 mmol/g protein and 0.4523 mmol/g protein, respectively. This reduction results from the Plastein reaction, which enzymatically reforms peptide bonds to recombine amino acids and peptide chains into larger molecules, thereby decreasing the content of free amino acids [36]. In contrast, when the hydrolysate is simply mixed with exogenous amino acids without undergoing the Plastein reaction, no chemical bonding break, and the amino acids remain in a free state, resulting in no distinct decrease in amino group level. Similar findings were declared by Li et al. [37], who demonstrated that the presence of the exogenous amino acid proline led to peptide condensation of sea cucumber protein hydrolysate after going through the Plastein reaction, causing altered chemical properties and a dramatic enhancement of angiotensin-converting enzyme (ACE) inhibitory activity.

### 3.2. Effect of Plastein Reaction Modification on Antioxidant Activity of All Samples

**Figure 1:**
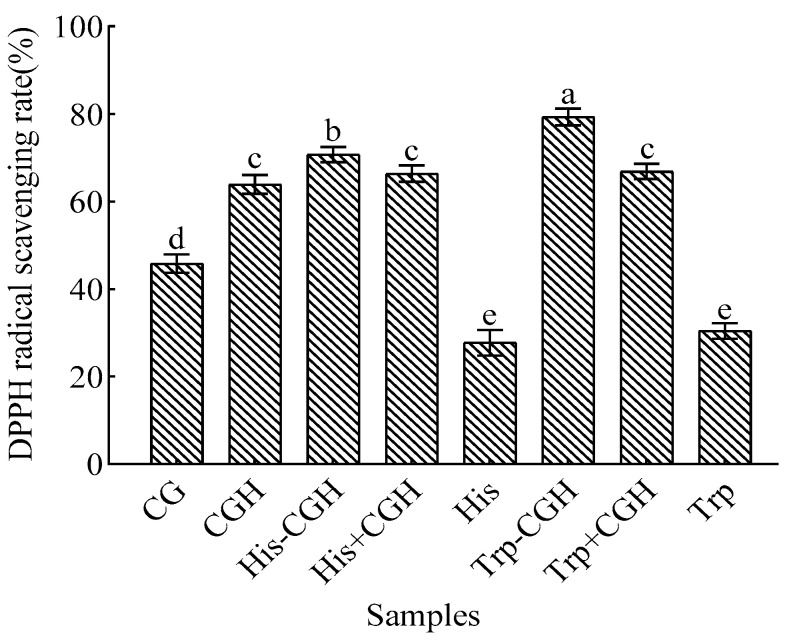
Effect of the Plastein reaction modification on DPPH radical scavenging activity of all samples. Note: One-way analysis of variance (ANOVA) was performed. Different letters in the figure indicate significant differences between groups in the analysis results (*p* < 0.05).

The results of the DPPH radical scavenging assay are shown in Figure 1. The data indicated that the DPPH radical scavenging activity of Trp-CGH (79.32%) was evidently higher (*p* < 0.05) than that of CG, CGH, and the physical mixture Trp+CGH, with increased values of 33.48%, 15.4%, and 12.44%, respectively, demonstrating its superior antioxidant capacity. The scavenging rate of His-CGH (70.73%) was clearly higher than that of His+CGH and CGH alone (4.39% and 6.81%, respectively) (*p* < 0.05). However, the DPPH radical scavenging rate of His-CGH (70.73%) was obviously lower than that of Trp-CGH (79.32%) (*p* < 0.05).

**Figure 2:**
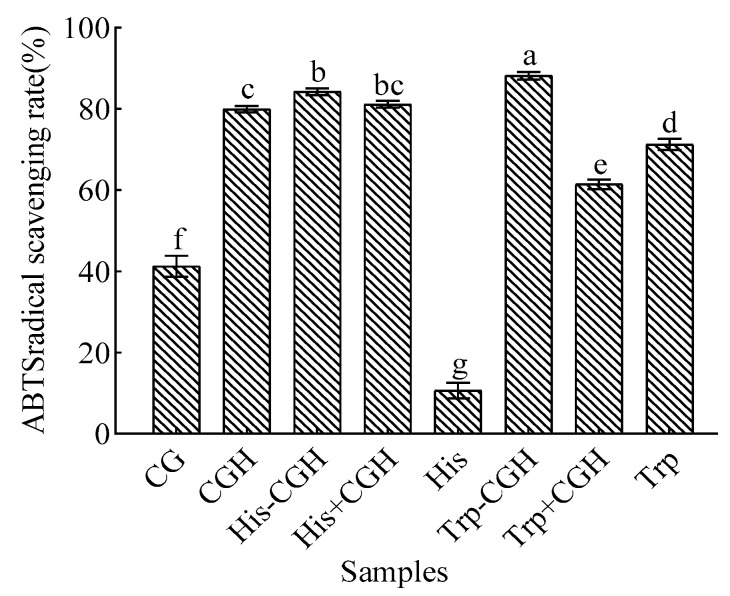
Effect of the Plastein reaction modification on ABTS radical scavenging activity of all samples. Note: One-way analysis of variance (ANOVA) was performed. Different letters in the figure indicate significant differences between groups in the analysis results (*p* < 0.05).

The results of the ABTS radical scavenging assay are shown in Figure 2. The ABTS free radical scavenging activity of Trp-CGH (88.19%) was significantly higher than that of CG (41.26%), CGH (79.93%), the physical mixture Trp+CGH (61.46%), and His-CGH (84.25%) (*p* < 0.05). Furthermore, the ABTS scavenging activity of His-CGH (84.25%) was significantly higher than that of CG (41.26%) and CGH (79.93%), and slightly higher than that of the physical mixture His+CGH (81.16%).

In summary, the modified compounds exhibited advantageous antioxidant activity. This suggests that the Plastein reaction is an effective method for reinforcing the antioxidant properties of protein hydrolysates, and the incorporation of exogenous amino acids can further raise the antioxidant activity of the modified products, in accordance with previous research. A previous study has confirmed that fish scale collagen hydrolysate was modified by the Plastein reaction through a combination of alkaline protease and flavourzyme, which evidently enhanced its antioxidant activity [38]. Compared with fish scale collagen hydrolysate, the hydroxyl radical scavenging rate of the Plastein reaction product increased by 21.1%. Casein hydrolysates modified by the Plastein reaction in the presence of one of three exogenous amino acids (leucine, valine, or phenylalanine) have also shown improved antioxidant activity [39]. Compared with casein hydrolysates, casein hydrolysates supplemented with Leu, Val, and Phe exhibited an obviously raised DPPH radical scavenging activity (8%, 7.8% and 9%, respectively) (*p* < 0.05). This enhancement may be due to structural changes in the peptide chains, reflecting the exposure of hydrophobic groups, and subsequent re-aggregation induced by the Plastein reaction [36]. The addition of exogenous amino acids through the Plastein reaction can further modulate the peptide chain structure, thereby increasing its capacity to interact with free radicals and neutralize them, which ultimately optimizes antioxidant activity.

### 3.3. Functional Characteristics Analysis of Modifiers

#### 3.3.1. Changes in Solubility of Modified Products

**Figure 3:**
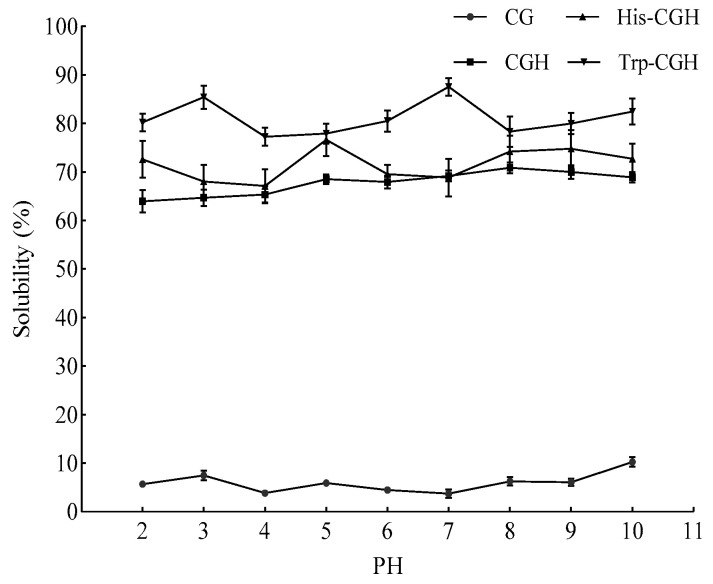
Solubility of all samples at different PH.

The solubility characteristics of the samples across pH 2–10 are presented in Figure 3. These data showed that Trp-CGH exhibited the highest solubility (87.53%) at pH 7, His-CGH reached its maximum solubility (76.59%) at pH 5, and CGH achieved the highest solubility (70.85%) at pH 8. Trp-CGH demonstrated higher solubility than CG, CGH, and His-CGH across the entire pH range of 2 to 10. The Plastein reaction reinforces the solubility of protein hydrolysates by promoting the coalescence of peptides into larger molecules through transpeptidation [36]. The incorporation of tryptophan enhances the polarity of the peptide chain and alters its spatial conformation, further elevating its solubility [40]. According to the research reported by Lazzari et al. [41], amino acid linkage via the Plastein reaction can modulate the interactions between polypeptide molecules, leading to increased solubility. Although the indole ring found in tryptophan is hydrophobic, its introduction may enhance solubility by optimizing charge distribution and spatial conformation.

#### 3.3.2. Changes in Emulsification and Emulsification Stability of Modified Products

**Figure 4:**
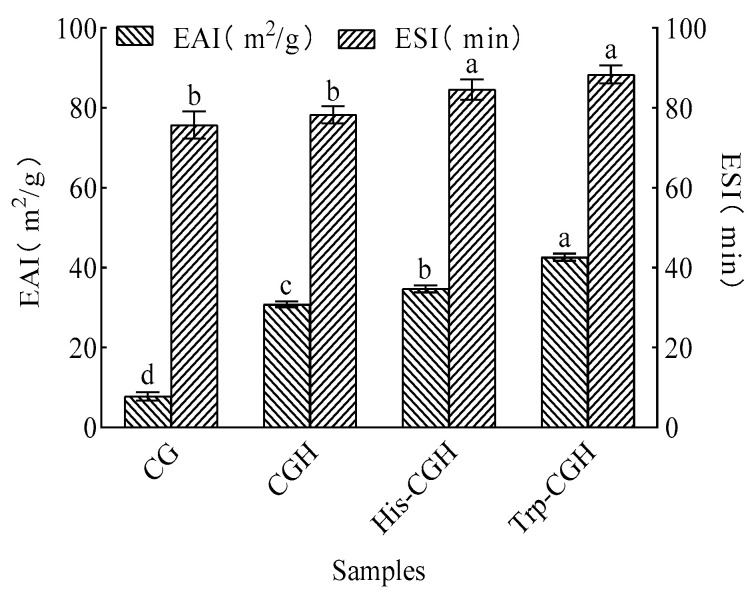
Effect of the Plastein reaction on emulsibility and emulsion stability of all samples. Note: One-way ANOVA was performed. Different letters in the figure indicate significant differences between groups in the analysis results (*p* < 0.05).

Figure 4 presents the emulsifying properties and emulsion stability of CG, CGH, His-CGH, and Trp-CGH. The emulsifying activity index (EAI) of Trp-CGH (42.59 m^2^/g) was prominently higher than that of His-CGH (34.70 m^2^/g), CGH (31.94 m^2^/g), and CG (7.738 m^2^/g) (*p* < 0.05). The emulsifying stability index (ESI) of Trp-CGH (89.96 min) and His-CGH (85.04 min) was not much different (*p* > 0.05), but both were obviously higher than those of CG (77.26 min) and CGH (79.39 min) (*p* < 0.05). These results indicate that the Plastein reaction can effectively enhance the emulsifying performance and emulsion stability of corn glutelin hydrolysate. The enhancement may contribute to increased surface hydrophobicity, potentially resulting from the aggregation of hydrophobic peptides, which can evidently influence emulsifying ability [42]. Zhou et al. [43] reported that silver carp protein hydrolysate (SCPH) exhibited clearly improved emulsifying properties through the Plastein reaction. The enhanced emulsifying performance reveals great potential for application in the food industry, such as when applied to ice cream, cream cakes, and similar products, it can help to texture smoother and more delicate.

#### 3.3.3. Effect of the Plastein Reaction on Foamability and Foam Stability of Modifiers

**Figure 5:**
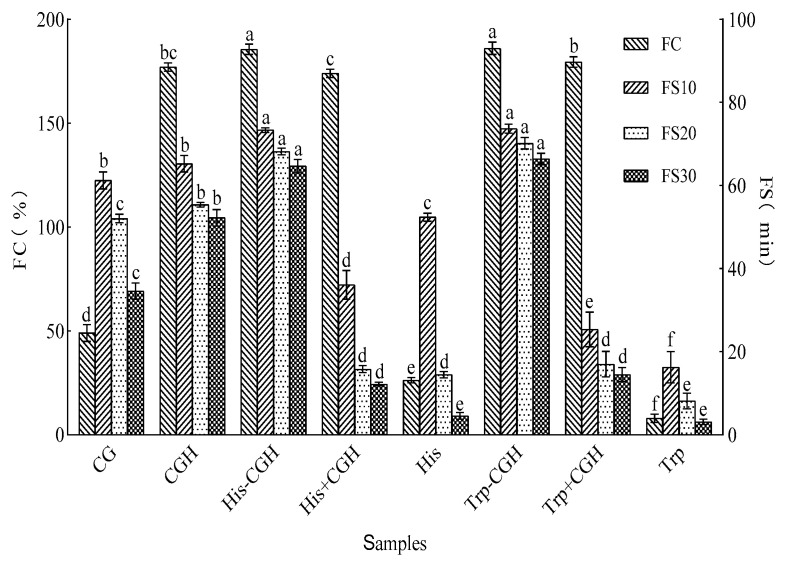
Effect of the Plastein reaction on foaming and foaming stability of all samples. Note: One-way ANOVA was performed. Different letters in the figure indicate significant differences between groups in the analysis results (*p* < 0.05).

Figure 5 displays the foaming properties and foaming stability of CG, CGH, His-CGH, and Trp-CGH. The foaming capacity (FC) of Trp-CGH and His-CGH was 186% and 185%, respectively, showing no visible difference between the two (*p* > 0.05), but both were sensibly higher than that of CG (49%), CGH (177%), His+CGH (174%), and Trp+CGH (179.5%) (*p* < 0.05). There were no distinct differences in foaming stability after 10 min (FS10, 73.66% and 73.32%), 20 min (FS20, 70.16% and 68.19%), and 30 min (FS30, 66.4% and 64.69%) between Trp-CGH and His-CGH (*p* > 0.05); however, foaming stability of the two modifiers was evidently higher than that of the other samples (*p* < 0.05). These results indicate that Trp-CGH and His-CGH exhibited impeccable foaming ability and foaming stability compared to other samples, suggesting that the Plastein reaction effectively optimizes the foaming properties of corn glutelin. The greatest advantage of the Plastein reaction is that it can recombine small peptides or amino acids, promote the formation of new peptide bonds, and reconstruct molecular conformations under protease catalysis, thereby effectively improving surface activity and enhancing foaming performance and foaming stability [36]. The introduction of tryptophan facilitates molecular adsorption at the gas–liquid interface and reduces surface tension, promoting bubble formation and strengthening foaming properties [44]. The hydrophobic grouping of the histidine side chain may reinforce hydrophobic interactions among protein molecules, leading to the formation of a more stable foam structure [45]. Additionally, the modified molecular structure may form a denser and more elastic interfacial film, effectively preventing bubble rupture and coalescence, thus heightening foam stability. This elevated foaming stabilization behavior highlights great potential for product applications requiring efficient foaming and stable foam structures. In the food industry, these modified products will be of great significance as safe and high-quality functional additive substitutes for baked products.

### 3.4. Effect of Modifier Addition on the Quality of Chiffon Cake

#### 3.4.1. Effect of Modifier Addition on the Density of Cake Batter

Batter density is a key measuring indicator in cake making, since it influences bubble formation and distribution, as well as the texture and sensory acceptability of the final product [46]. Table 2 shows the effect of adding different modifiers on the density of cake batter. The batter density with the Trp-CGH sample was obviously lower than that of the control (*p* < 0.05), with a reduction of 0.028 g/cm^3^. Compared with the control group, the batter density with the His-CGH sample exhibited a decrease in batter density of 0.021 g/cm^3^, but this difference was not statistically significant (*p* > 0.05). The reduction in cake batter density can be primarily owing to the excellent foaming properties of His-CGH and Trp-CGH, which enable protein to encapsulate and stabilize air during the mixing process. The formation of foam increases the volume of gas within the batter, thereby reducing the relative proportions of liquid and solid components and lowering the overall batter density [47]. Jyotsna et al. reported that the partial replacement of flour in egg-free cake with whey protein concentrate (WPC) resulted in decreased batter density, a finding consistent with the results of this study [48].

#### 3.4.2. Effect of the Addition of Modifiers on Physical Characteristics of Chiffon Cake

**Figure 6:**
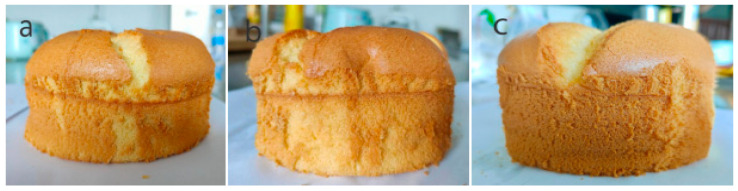
The effect of adding His-CGH and Trp-CGH on the overall appearance of the chiffon cake. (**a**) Control sample, (**b**) cake sample with His-CGH, and (**c**) cake sample with Trp-CGH.

Specific volume and baking loss rate are major quality parameters of baked products. The specific volume and baking loss rate of the cakes are summarized in Table 2. The results show that the control cake had the lowest specific volume (2.63 cm^3^/g) and the highest baking loss rate (8.07%). The addition of His-CGH and Trp-CGH in chiffon cake significantly increased the specific volume of the cake (2.86 cm^3^/g and 2.92 cm^3^/g, respectively) and significantly decreased the baking loss rate (7.25% and 7.08%, respectively) (*p* < 0.05). As shown in Figure 6, compared with the control group, the volume of the cake samples added with His-CGH and Trp-CGH significantly increased; meanwhile, the overall appearance of the cake samples was firmer and more stable. These improvements may be by reason of the enhanced foaming ability and foam stability of the modified proteins. Proteins with good foaming properties can generate more and more stable bubbles, which will expand during the baking process and contribute to a greater cake volume and higher specific volume [49]. Such proteins can reduce water loss during the baking process, thus diminishing the baking loss rate [50]. Camargo et al. [51] displayed that adding different proportions of whey protein to banana cake remarkably increased the specific volume of banana cake and decreased the baking loss rate of banana cake, compared with the control group. Subagio et al. [52] discovered that the 1% chickpea protein isolate addition in cakes could obviously reduce the baking loss rate to 15.50% and increase the specific volume to 2.63 cm^3^/g. The corresponding detection indicators for the control group were 17.25% and 2.17 cm^3^/g, respectively. These results fully confirm that a protein with complete functional properties can optimize the physical characteristics and the quality of cake samples.

**Figure 7:**
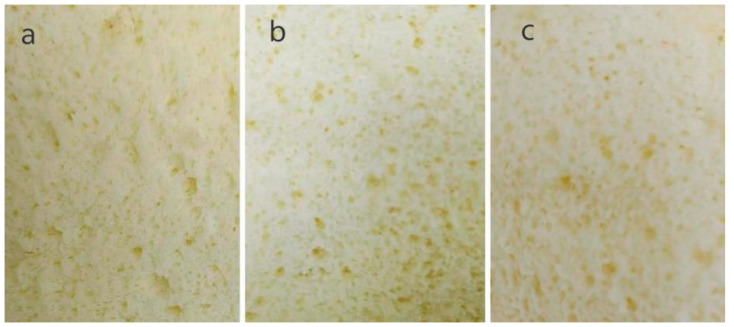
Effect of His-CGH and Trp-CGH addition on the texture of chiffon cake crumb. (**a**) Control sample, (**b**) cake sample with His-CGH, and (**c**) cake sample with Trp-CGH.

Porosity is a critical influencing factor that affects the texture of baked products, with higher porosity generally resulting in a lighter and fluffier structure. Table 2 displays the porosity of the different cake samples. Compared with the control group, the His-CGH and Trp-CGH groups exhibited significantly higher porosity (3.48% and 4.41%, respectively) (*p* < 0.05). This improvement may be attributed to the raised emulsifying and foaming properties of the modified proteins. During the process of cake making, the air is introduced into the batter by beating, and the protein can be adsorbed on the surface of the bubbles to form a stable interface film to prevent the coalescence and rupture of the bubbles, thereby improving the porosity [53]. Rahim et al. [31] reported that the addition of sesame protein isolate (SPI) and transglutaminase (TG) to the cake recipe increased porosity. The effect of adding His-CGH and Trp-CGH on the texture of chiffon cake is illustrated in Figure 7. The small, evenly distributed pores found in the cake sample with modifiers may obviously indicate a fine and uniform texture. Xiao et al. [54] showed that the chiffon cake supplemented with a ferulic acid–egg white protein covalent complex displayed uniform and dense bubble pore size, which was consistent with the results of this experiment.

#### 3.4.3. Effect of the Addition of Modifiers on Cake Color

The effect of the modifier addition on the color of the cake crust and crumb is presented in Table 3. The lightness (L) values of both the crust and crumb color of cakes containing His-CGH and Trp-CGH were significantly lower than those of the control (*p* < 0.05). The red-green color component (a) of both crust and crumb showed a slight decrease, but no significant difference was observed (*p* > 0.05). For the yellow-blue color component (b), the crust color of the cake sample consisting of the His-CGH showed an obvious decrease, while that of the cake sample including the Trp-CGH sample decreased slightly without reaching statistical significance. In contrast, the b values in the inner crumb of both modified samples significantly reduced. These results indicate that the addition of the modifier resulted in a darker crust color, compared to the control. The classic Maillard reaction occurs under high-temperature conditions during the baking process, and amino compounds are key factors affecting the Maillard reaction, thus leading to the formation of brown or dark-colored compounds. The addition of these modifiers promotes the Maillard reaction process, thereby reducing the brightness of the cake crust [55]. Subagio et al. [52] investigated the effects of lentil protein isolate on cake baking characteristics and found that its addition of modifiers endowed the cake with a darker crust, which is coincidence with the findings of this study.

#### 3.4.4. Effects of the Addition of Modifiers on the Texture Characteristics of Cake Samples

The texture profile analysis (TPA) results of cake samples are summarized in Table 4. Compared with the control group, the hardness of the cake sample containing Trp-CGH sharply reduced (0.36) (*p* < 0.05), while the hardness of the cake sample with the His-CGH decreased (0.27) without obvious statistical significance (*p* > 0.05). The springiness of both His-CGH- and Trp-CGH-modified cakes clearly increased (1.05 ± 0.35 and 0.97 ± 0.55, respectively) (*p* < 0.05), and the chewiness also increased (1.72 ± 1.01 and 2.14 ± 1.16, respectively). No significant differences were observed in cohesiveness between the control and the modified samples (*p* > 0.05). These changes may be attributed to the excellent foaming properties and foam stability of the modifiers, which can obviously affect the hardness, elasticity, chewiness, and cohesiveness of the cake. The results assessed by Rahim et al. demonstrated that the addition of sesame protein isolate (SPI) and transglutaminase (TG) could reduce the hardness of the cake. For all samples, the hardness value of the control group was the highest (1.06 N), and the hardness of the cake with 3% SPI and 0.6% TG was 0.54 N [31]. Gularte et al. [56] showed that the incorporation of different legumes (lentil and bean) into a gluten-free cake sample can significantly increase the elasticity of the cake. The addition of lentil and bean to gluten-free cakes clearly increased the chewiness of the cake sample. Similar results provided by Tomic et al. [57] revealed that the hardness of gluten-free bread samples with millet flour decreased, and the substitution of millet flour caused a complete loss of bitter taste derived from millet. Adding functional proteins with excellent physical and chemical properties to cake recipe products during the baking process can help to form a stable bubble structure and maintain their shape, thereby improving the overall appearance of the baked product.

#### 3.4.5. Effect of the Addition of Modifiers on Sensory Quality of Cake Samples

**Figure 8:**
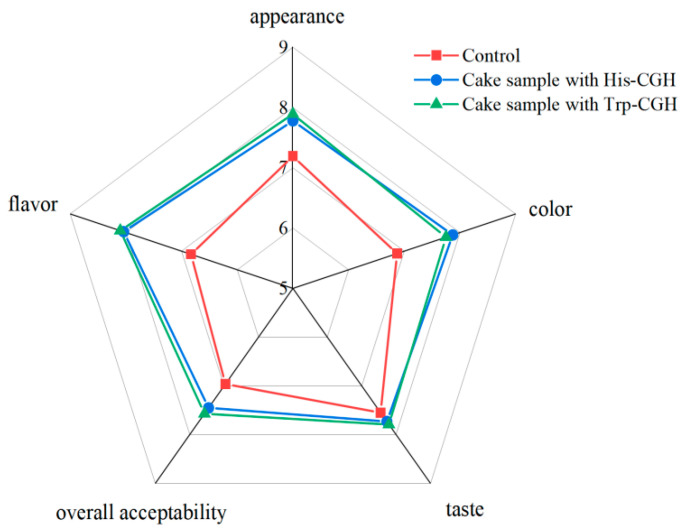
Graphical representation of sensory analysis of the cake samples. Note: Control: Cake sample without added modifier; His-CGH: cake supplemented with His-CGH; Trp-CGH: cake supplemented with Trp-CGH.

In this study, the sensory evaluation of the cake was conducted based on five attributes—appearance, flavor, taste, color, and overall acceptability—and presented in Figure 8. The results indicated that when the overall sensory score of a sample exceeded 5 points, it reflected good sensory acceptability. The cake samples supplemented with His-CGH and Trp-CGH significantly received higher scores in flavor, increasing by 1.21 ± 0.76 and 1.27 ± 0.69, respectively, compared to the control (*p* < 0.05). Moreover, the cake samples containing His-CGH received higher scores in appearance, taste, and color; compared to the control, the resultant sensory scores increased by 0.59, 0.18, and 1.00, respectively. The cake samples supplemented with Trp-CGH received higher scores in appearance, taste, and color; compared to the control, the resultant sensory scores increased by 0.70, 0.24, and 0.68, respectively (*p* > 0.05). The cake samples, including His-CGH and Trp-CGH, received higher scores in overall acceptability, compared to the control, increasing by 0.49 and 0.61, respectively. The enhanced sensory acceptability of the cake samples may be attributed to elevated foam stabilization, increased specific volume and porosity, and improved texture. Recent studies have shown that the addition of plant-based proteins with good foaming and foam-stabilizing properties can apparently affect the sensory acceptability of baked products; accordingly, the incorporation of proteins with excellent foaming properties can enhance texture and taste, thereby improving overall acceptability [58]. The results of another literature study indicated that adding different proportions of beetroot powder to the cake sample could improve cake acceptance, and the cake sample with 15% beetroot powder was more popular than the other groups [59]. Suresh et al. [60] demonstrated that the sensory scores for biscuit recipe powder with soy protein isolate and whey protein isolate were higher than those of both the control group and biscuit recipe powder with pea protein isolate. Shin et al. [61] identified that the addition of soy protein isolate alone or in combination with TG significantly affected the sensory acceptance of gluten-free rice bread.

## 4. Conclusions

In this study, corn glutelin was hydrolyzed by alkaline protease, and then the corresponding hydrolysate was modified via the Plastein reaction under conditions of sufficient exogenous amino acids (histidine and tryptophan). The resulting modifiers, His-CGH and Trp-CGH, exhibited excellent emulsifying stability, foaming capacity, and foaming stability. Cake recipe product with His-CGH and Trp-CGH exhibited reduced batter density, increased foaming capacity, and improved emulsifying stability. Furthermore, the incorporation of these modified peptides influenced the color, texture, and overall quality of the cakes, leading to enhanced specific volume, reduced hardness, and quality improvement in their taste and sensory acceptability.

This study represents the first attempt to modify corn glutelin hydrolysate through the Plastein reaction, thereby providing a theoretical basis for the functional modification of corn glutelin. In addition, the Plastein reaction-modified corn glutelin hydrolysate was innovatively applied to chiffon cake formulation to investigate its potential in improving cake quality and functionality. This application opens up new possibilities for utilizing Plastein reaction-modified corn glutelin hydrolysate in chiffon cake and other baked products. The findings of this research hold significant application potential in the food processing fields and also offer a novel strategy for the high-value utilization of plant proteins.

## Figures and Tables

**Table 1:** Free amino content of different samples.

	Samples	–NH2 Content (mmol/g Protein)
CG	corn glutelin	0.0231 ± 0.0013 f
CGH	corn glutelin hydrolysate	1.7928 ± 0.0170 c
His-CGH	The Plastein modifier of CGH and His	1.5724 ± 0.0150 d
His+CGH	The mixture of CGH and His	1.9523 ± 0.0160 a
Trp-CGH	The Plastein modifier of CGH and Trp	1.4382 ± 0.0120 e
Trp+CGH	The mixture of CGH and Trp	1.8916 ± 0.0100 b

Note: Experimental results are expressed as mean ± standard deviation (SD), n = 3. One-way analysis of variance (ANOVA) was used, and different lowercase letters (a–f) superscripted in the same column indicate significant differences between groups (*p* < 0.05).

**Table 2:** Effect of the addition of modifiers on the physical characteristics of cakes.

Samples	Specific Volume (cm^3^/g)	Baking Loss (%)	Porosity (%)	Batter Density (g/cm^3^)
C	2.63 ± 0.12 ^b^	8.07 ± 0.17 ^a^	27.47 ± 0.63 ^b^	0.581 ± 0.012 ^a^
C_1_ (His-CGH)	2.86 ± 0.10 ^a^	7.25 ± 0.13 ^b^	30.95 ± 0.82 ^a^	0.560 ± 0.012 ^ab^
C_2_ (Trp-CGH)	2.92 ± 0.06 ^a^	7.08 ± 0.24 ^b^	31.88 ± 0.52 ^a^	0.553 ± 0.013 ^b^

Note: Experimental results are expressed as mean ± standard deviation (SD), n = 3. One-way analysis of variance (ANOVA) was used, and different lowercase letters (a and b) superscripted in the same column indicate significant differences between groups (*p* < 0.05). C: Control cake; C_1_ (His-CGH): Cake made by adding His-CGH; C_2_ (Trp-CGH): Cake made by adding Trp-CGH.

**Table 3:** Effect of the addition of modifiers on the color of the cake samples.

Type of Cake		C	C_1_ (His-CGH)	C_2_ (Trp-CGH)
Crust color	L	50.34 ± 1.60 ^a^	40.77 ± 1.46 ^b^	39.42 ± 1.55 ^b^
	a	12.68 ± 1.51 ^a^	10.58 ± 0.88 ^a^	11.61 ± 1.12 ^a^
	b	25.21 ± 1.75 ^a^	20.07 ± 2.59 ^b^	23.14 ± 1.75 ^ab^
Crumb color	L	30.40 ± 2.38 ^a^	19.63 ± 0.63 ^b^	14.79 ± 2.36 ^c^
	a	2.15 ± 0.84 ^a^	1.45 ± 0.53 ^a^	1.28 ± 0.42 ^a^
	b	19.65 ± 1.66 ^a^	15.79 ± 0.51 ^b^	11.14 ± 1.51 ^c^

Note: Experimental results are expressed as mean ± standard deviation (SD), n = 3. One-way analysis of variance (ANOVA) was used, and different lowercase letters (a–c) superscripted in the same column indicate significant differences between groups (*p* < 0.05). C: Control cake sample; C_1_ (His-CGH): Cake sample with His-CGH; C_2_ (Trp-CGH): Cake sample with Trp-CGH.

**Table 4:** Effect of the addition of modifiers on the texture characteristics of cakes.

Samples	Hardness (N)	Springiness (mm)	Chewiness (N)	Cohesion (1)
C	2.37 ± 0.14 ^a^	4.46 ± 0.32 ^b^	9.35 ± 0.45 ^b^	0.70 ± 0.02 ^a^
C_1_ (His-CGH)	2.10 ± 0.16 ^ab^	5.51 ± 0.13 ^a^	11.07 ± 0.90 ^a^	0.69 ± 0.01 ^a^
C_2_ (Trp-CGH)	2.01 ± 0.12 ^b^	5.43 ± 0.45 ^a^	11.49 ± 1.07 ^a^	0.69 ± 0.02 ^a^

Note: Experimental results are expressed as mean ± standard deviation (SD), n = 3. One-way analysis of variance (ANOVA) was used, and different lowercase letters (a and b) superscripted in the same column indicate significant differences between groups (*p* < 0.05). C: Control cake sample; C_1_ (His-CGH): cake sample endowed with His-CGH; C_2_ (Trp-CGH): cake sample endowed with Trp-CGH.

## Data Availability

The original contributions presented in this study are included in the article. Further inquiries can be directed to the corresponding authors.
